# A case for a bigger role of transcutaneous vagus nerve stimulation in brain injury rehabilitation

**DOI:** 10.3325/cmj.2025.66.241

**Published:** 2025-06

**Authors:** Martina Miklić Bublić, Dinko Tonković, Vilena Vrbanović Mijatović

**Affiliations:** 1 University Department of Anesthesiology, Resuscitation and Intensive Care, Zagreb University Hospital Center, Zagreb, Croatia; 2 Department of Anesthesiology, Reanimatology and Surgical Intensive Care Medicine, University of Zagreb, School of Medicine, Zagreb, Croatia *mmiklic@kbc-zagreb.hr*

## Acute brain injury 

Acute brain injury is among the leading health care issues. It is characterized by high mortality and morbidity, limited therapeutic options, prolonged hospital stay, and disabilities that undermine the quality of life and increase health care costs. Brain injury can be traumatic or non-traumatic. The incidence of traumatic brain injury (TBI) globally is 27 to 69 million cases per year, and the prevalence is 55.5 million, mainly due to falls in high-income countries and traffic accidents in low-income countries (1). Its global socio-economic impact is huge, around US $400 billion dollars per year (2). Among the most challenging non-traumatic brain injuries stands aneurysmal subarachnoid hemorrhage (aSAH), with an incidence of 10-15 per 100 000 patients annually (3). The financial burden varies throughout the world. The cost of hospitalization for an aSAH patient in the United States is $65 900 (3). The overall mean cost of treatment in neurocritical ICU per patient in Finland is €45,799, with higher treatment costs for SAH than for TBI (4). The treatment costs for a patient with SAH is €61 243 in Canada and €82 546 in the UK (4).

In the treatment of primary and secondary brain injury, both surgical and non-surgical procedures are important. Recent studies show that neuromodulation by vagus nerve stimulation (VNS) may contribute to better outcomes in brain- trauma patients (5,6).

## Evidence supporting the use of transcutaneous VNS in brain injury rehabilitation

VNS was first used in the clinical setting as an invasive procedure for the treatment of pharmacoresistant epilepsy. Later, noninvasive transcutaneous VNS developed as a safer approach for treating cerebral edema, epileptic seizures, and blood-brain barrier disruption, for facilitating the recovery of motoric and cognitive functions, and for immunomodulation (5). Transcutaneous VNS efficiency in brain injury treatment may be explained by several mechanisms (5). According to the noradrenergic hypothesis, VNS upregulates endogenous noradrenergic activity due to the activation of the locus coeruleus, the release of noradrenaline in the amygdala, and a higher overall concentration of noradrenaline in the brain (5). Accordingly, the rise in cerebral perfusion pressure enables better brain tissue oxygenation and reperfusion of the penumbra zone. Noradrenaline suppresses seizures by protecting GABAergic neurons and decreasing neuronal hyper-excitability. It also acts anti-inflammatory on microglia cells, lowering cytokine and chemokine production (5). Wu et al have recently shown that transcutaneous VNS significantly modulates serum inflammatory cytokines in sepsis patients (7). Another anti-inflammatory mechanism is the interaction of VNS with peptide hormone ghrelin, which lowers tumor necrosis factor (TNF)-alpha levels after brain injury (5). Under vagal stimulation, acetylcholine reduces pro-inflammatory cytokines, such as TNF, interleukin (IL)-1 β, IL-6, and IL-18 (6). At the same time, it reduces the pro-inflammatory M1 phenotype of microglia, which promotes oxidative stress and mitochondrial impairment, in favor of the neuroprotective M2 phenotype (6). Transcutaneous may alleviate cognitive dysfunction, as it reduces neuroinflammation and enhances memory function (6) by the stimulation of peripheral and central cholecystokinin receptors (6). This implicates the involvement of the brain-gut axis, as transcutaneous VNS could balance the inflammatory microenvironment of the intestinal flora (6). Furthermore, VNS associated with rehabilitation therapy significantly increases functional motor recovery (8), which is particularly important for the activities of daily living and better quality of recovery. Motor recovery relies on the neuroplasticity of the brain, which is promoted by neuromodulators such as noradrenaline, acetylcholine, and serotonine (8).

## The prevailing practice in Croatia

To the best of our knowledge, no reports have been published so far on the use of transcutaneous VNS in clinical settings in Croatia. However, the limited possibilities of the present therapies in ICU settings, especially therapies for neurocritical ICU patients, have opened up space for new noninvasive methods. As a result, worldwide interest in transcutaneous VNS is growing due to its role in enhancing neuroplasticity and preventing systemic inflammation. Transcutaneous VNS has already been approved in Europe for the treatment of epilepsy (5). Recent studies have shown its safety in patients with SAH and stroke (9), and a significant improvement in the functional recovery of patients in minimally conscious state following transauricular transcutaneous VNS lasting for four weeks (10).

Given these results, we plan to investigate the role of transcutaneous VNS in patients with brain injury in preventing systemic inflammatory response and seizures, and promoting neuromodulation and cognitive and motoric recovery, with the emphasis on the early phase of hospitalization and ICU stay. Currently, there are no specific guidelines on the length or frequency of transcutaneous VNS. It is still unclear when it is the best time to start transcutaneous VNS treatment, so we plan to compare the patients in whom stimulation is begun right after ICU admission and those in whom it is started after one week following brain injury. We also plan to compare the outcomes between traumatic and non-traumatic brain injury after transcutaneous VNS.

## Future perspectives

Transcutaneous VNS offers a safe and non-invasive approach to patients with brain injury, and can be easily administered. It has the potential to reduce mortality and morbidity and enhance patient outcomes, as parasympathetic stimulation via the vagus nerve benefits many organ systems. Should the results of our study justify the use of transcutaneous VNS in the neurocritical ICU, we could implement it as a noninvasive therapeutic method in everyday care to reduce the length of hospital stay and high costs of ICU treatment. However, further studies are required to inform the best guidelines and protocols for the administration of transcutaneous VNS.
